# The emerging burden of liver disease in cystic fibrosis patients: A UK nationwide study

**DOI:** 10.1371/journal.pone.0212779

**Published:** 2019-04-04

**Authors:** M. B. Toledano, S. K. Mukherjee, J. Howell, D. Westaby, S. A. Khan, D. Bilton, N. J. Simmonds

**Affiliations:** 1 MRC-PHE Centre for Environment and Health, School of Public Health, Imperial College London, London, United Kingdom; 2 Liver Unit, Imperial College London, St. Mary’s Hospital, London, United Kingdom; 3 Burnet Institute Centre for Public Health, St. Vincent’s Hospital, Melbourne, Victoria, Australia; 4 Department of Gastroenterology, Hammersmith Hospital, London, United Kingdom; 5 Adult Cystic Fibrosis Centre, Royal Brompton Hospital, London, United Kingdom; 6 National Heart and Lung Institute, Imperial College, London, United Kingdom; Medizinische Fakultat der RWTH Aachen, GERMANY

## Abstract

**Objective:**

Cystic fibrosis associated liver disease (CFLD) is the third largest cause of mortality in CF. Our aim was to define the burden of CFLD in the UK using national registry data and identify risk factors for progressive disease.

**Methods:**

A longitudinal population-based cohort study was conducted. Cases were defined as all patients with CFLD identified from the UK CF Registry, 2008–2013 (n = 3417). Denominator data were derived from the entire UK CF Registry. The burden of CFLD was characterised. Regression analysis was undertaken to identify risk factors for cirrhosis and progression.

**Results:**

Prevalence of CFLD increased from 203.4 to 228.3 per 1000 patients during 2008–2013. Mortality in CF patients with CFLD was more than double those without; cirrhotic patients had higher all-cause mortality (HR 1.54, 95% CI 1.09 to 2.18, p = 0.015). Median recorded age of cirrhosis diagnosis was 19 (range 5–53) years. Male sex, Pseudomonas airway infection and CF related diabetes were independent risk factors for cirrhosis. Ursodeoxycholic acid use was associated with prolonged survival in patients without cirrhosis.

**Conclusions:**

This study highlights an important changing disease burden of CFLD. The prevalence is slowly increasing and, importantly, the disease is not just being diagnosed in childhood. Although the role of ursodeoxycholic acid remains controversial, this study identified a positive association with survival.

## 1. Introduction

Cystic fibrosis (CF) is the most common autosomal recessive disorder in Europe, affecting 1 in 2500 live births in the UK annually[1]. It is one of the commonest life-shortening genetic diseases in the Western world: however, prognosis has improved markedly with modern multidisciplinary care: median predicted survival in the UK is currently 47.0 years[2]. This is primarily due to improved management of respiratory and nutritional complications of CF. As a result of this, other non-pulmonary complications are becoming increasingly important[3,4]. Hepatic complications (encompassing cirrhosis, portal hypertension with variceal bleeding and ultimately liver transplantation) are an increasingly important cause of morbidity and mortality[5,6].

It is estimated that between 20 and 40% of all CF patients have associated liver disease[7–9]. Although clinically heterogeneous, approximately 5–10% of CF patients develop multilobar cirrhosis in the first decade of life[8,9]. A proportion of these progress to portal hypertension and consequent sequelae, including variceal bleeding, necessitating repeated intervention and liver transplantation. CF associated liver disease (CFLD) is recognized as the third largest cause of mortality in CF following respiratory disease and complications of lung transplantation[10,11]. Risk factors for the development of liver disease within CF patients have been described[12] including an association with the SERPINA1 Z allele, a polymorphism in the alpha-1-antitrypsin gene[13]. Early diagnosis is complicated by a long sub-clinical phase and the lack of a reliable screening tool: changes in liver function tests and ultrasonographic appearances can be variable and temporary. Furthermore, although ursodeoxycholic acid (UDCA) is commonly prescribed, there is no proven effective treatment prior to liver transplantation. Established liver cirrhosis remains a barrier to lung transplantation in patients with severe pulmonary disease; thus a better understanding of CFLD and new strategies to prevent progressive liver damage is therefore a priority. The majority of published studies are single centre and include mainly paediatric cases[5,7–9,12,14]. Larger recently published studies have focussed on subsets of patients within the spectrum of CFLD, such as those with portal hypertension[15].

The aim of this study was to evaluate for the first time the burden of all CFLD across the UK, identify possible risk factors for the development of cirrhosis, and ascertain the effectiveness of UDCA treatment.

## 2. Methods

### 2.1 Study population and design

The study population comprised all patients on the UK CF Registry identified with liver disease between 2008 and 2013. The UK Cystic Fibrosis Registry, with records of over 10,000 patients, represents 99% of the UK CF population. Data is collected annually at a time of clinical stability and includes information from key domains (demographics, diagnosis, complications [which includes liver disease], mortality, laboratory data, nutrition, pulmonary function, respiratory microbiology and physiotherapy). Further details of the registry can be found in its data resource profile[1]. ‘Liver disease’ as defined on the UK CF Registry includes the following categories:

1. Patients with elevated liver enzymes (alanine aminotransferase ALT, aspartate aminotransferase AST or gamma glutamyltransferase GGT).
2. Patients with abnormal (but non-cirrhotic) appearances at liver ultrasound, including steatosis.
3. Patients with evidence of cirrhosis at liver ultrasound.
4. Patients with evidence of cirrhosis and portal hypertension as evidenced by the presence of splenomegaly, ascites or gastro-oesophageal varices.

We undertook a longitudinal population-based cohort study. Cases were thereafter split into ‘non-cirrhotic’ cases by combining categories 1 and 2 and ‘cirrhotic’ cases, combining categories 3 and 4. This is in broad concordance with recently agreed phenotypic categories of CFLD[16].

Clinical and demographic data taken from the Registry were recorded for each patient with liver disease including forced expiratory volume in 1 second (FEV_1_), forced vital capacity (FVC), CF genotype, body mass index (BMI), liver disease category, CF-related diabetes (CFRD), gastrointestinal and hepatological complications, airway infection with *Pseudomonas aeruginosa*, and select socioeconomic indicators. ‘Chronic’ airway infection status required at least three isolates of *P*.*aeruginosa* in sputum collected over the previous year.

It is a standard of care in the UK for all patients with CF to have annual liver function tests and alternate year abdominal ultrasound examinations (for children) to monitor liver disease status and progression. Therefore, if a patient does show signs of liver progression this will be captured in the Registry.

Sequential annual data were used to calculate CFLD prevalence, annual and cumulative incidence, and mortality rates per 1000 CF patients. Denominator data were derived from the entire UK CF Registry dataset (all patients with complete records registered each year during the study period). Clinical variables associated with cirrhosis, progression to cirrhosis during the study period and overall survival were then determined.

### 2.2 Statistical methods

Normally distributed continuous data were described using means and standard deviation and compared using the Student t-test. Non-normally distributed data were described using medians and interquartile range and compared using Wilcoxon rank-sum and Kruskall-Wallis tests. Categorical data were compared using Chi square tests.

Logistic regression was used in order to identify associations with the presence of cirrhosis. Thereafter, longitudinal analysis was utilised in order to identify those variables that were associated with progression of disease during the study period. This is because there is a well observed group of patients with a more aggressive form of CFLD in whom disease progresses rapidly, usually early in life. We also wished to use the dataset to attempt to identify variables associated with overall survival.

Multiple logistic regression analysis was performed with stepwise model building. Likelihood ratio testing was performed with each variable subtraction. Variables with p-values of 0.05 or less on univariate analysis were included in the initial model, as well as variables considered to be clinically relevant based on published data. “Goodness-of-fit” for the final model was assessed by Hosmer-Lemeshow test. Odds ratios and 95% confidence intervals were calculated using logistic regression after converting continuous variables to categorical based on quartiles (age, weight, height) or clinically relevant categories (BMI, FEV1).

Time-to-event analyses were performed for all-cause mortality and progression to cirrhosis, initially using log-rank testing for univariate analysis. Multivariate analysis of variables independently associated with survival and time to disease progression (with a p-value <0.01) was performed using Cox proportional hazards modelling. Likelihood ratio testing was determined with each variable subtraction from the model and proportional hazards assumption testing was performed using Schoenfeld residuals.

All statistical analyses were performed using STATA version 12.1 (Statacorp, Texas USA). NHS research ethics approval was granted for the collection of data into the UK database. The UK Cystic Fibrosis Registry Steering Committee approved the use of anonymised data for this study.

## 3. Results

### 3.1 CFLD incidence, prevalence & mortality: 2008 to 2013

3417 individual cases of CFLD were recorded, with a total of 10,366 person-years of follow-up (median follow up time 4 years, IQR 3–6 years, range 1–6 years). The majority (89.7%) had non-cirrhotic liver disease. 55.5% of cirrhotic cases had portal hypertension (Table 1). A summary of socio-economic variables in the cohort is outlined in S1 Table.

**Table 1:** Summary of the distribution of demographic and clinical variables in the whole study cohort (n = 3417).

Clinical Variable	Number (%)/ Mean +/- SD
Adult	2355 (68.9%)
Child (<16yrs)	1062 (31.1%)
Age (n = 3417)	21.3 years ±11.5 years
Gender (n = 3417)	
Male	2003 (58.6%)
Female	1414 (41.4%)
Stage of liver disease (n = 3417)	
Non-cirrhotic liver disease	3064 (89.7%)
Cirrhosis without portal hypertension	157 (4.6%)
Cirrhosis with portal hypertension	196 (5.7%)
BMI (n = 3341)	
<18.5kg/m^2^	1122 (33.6%)
18.5-25kg/m^2^	1811 (54.2%)
>25kg/m^2^	408 (12.2%)
FEV1 (%) (n = 2946)	
>80%	1086 (36.9%)
61–80%	824 (28.0%)
40–60%	585 (19.9%)
<40%	451 (15.3%)
Ursodeoxycholic acid therapy (n = 3271)	1749 (53.5%)
Pancreatic enzymes (n = 3325)	3135 (94.3%)
Gallbladder complications (n = 3417)	25 (0.7%)
Gastro-oesophageal reflux (n = 3417)	500 (14.6%)
Intestinal obstruction (n = 3417)	237 (6.9%)
CF-related diabetes (n = 3417)	916 (26.8%)
Variceal bleeding (n = 3417)	13 (0.4%)
Chronic *P*. *aeruginosa* infection (n = 3417)	
No	1368 (40.0%)
Yes	2049 (60.0%)

There were 2180 incident cases of CFLD diagnosed, giving an incidence rate of 63.5 cases per 1000 CF patients. Annual incidence of CFLD varied during the study period from 89.2 cases of CFLD per 1000 CF patients in 2009 to 53.4 in 2013. Annual prevalence of CFLD increased from 203.4 to 228.3 cases per 1000 CF patients between 2008 and 2013 (Table 2).

**Table 2:** Annual incidence and prevalence of CF liver disease.

Year	Total CF population*	Annual total CF patient population at risk for developing CFLD**	Annual number of new CFLD cases	Incidence of CFLD (per 1000 CF patients; ± 95% CI)	Annual number CFLD cases	Prevalence of CFLD (per 1000 CF patients; ± 95% CI)
2008	6082	N/A	1237	N/A	1237	203.4 (198.8–216.2)
2009	7378	6427	573	89.2 (82.3–96.2)	1527	207.0 (197.8–216.3)
2010	7938	6701	384	57.3 (51.7–62.9)	1619	204.0 (194.8–213.2)
2011	8680	7226	484	67.0 (61.2–72.8)	1938	223.3 (214.5–232.1)
2012	8794	7187	364	50.7 (45.6–55.8)	1978	224.1 (215.4–232.8)
2013	9052	7018	375	53.4 (48.1–58.7)	2067	228.3 (219.7–236.9)
**Overall incidence rate (2009–2013) per 1000 CF patients**				**63.5 (60.9–66.1)**		

*: Total CF population = number of patients on the UK CF registry with complete records

**: Population at risk of CFLD = Total CF population—known registry cases of CFLD

Annual incidence of cirrhosis cases increased from 12.2 to 16.0 cases per 1000 CFLD patients between 2009 and 2013. Annual prevalence of cirrhosis cases fell from 157.6 to 137.9 cirrhosis cases per 1000 CFLD patients between 2008 and 2013.

Two hundred deaths in patients with CFLD were recorded during the study period: five children (2.5%) and 195 adults (97.5%). This represented 5.9% of cases in which mortality data were known. Eight of those patients (4%) died of liver-related complications. Annual and total crude mortality rates were higher in CFLD than CF: 19.3 deaths per 1000 CF patients per year were recorded in those with CFLD, compared with 7.6 deaths per 1000 CF patients per year in those without (S2 Table).

### Clinical associations with cirrhosis

Given the progressive natural history of liver cirrhosis, age is an important risk factor. The analysis of variables associated with the presence of cirrhosis in CFLD was therefore stratified into children (<16 years old) and adults. Summaries of the distribution of clinical variables with significant differences between cirrhotic and non-cirrhotic CFLD are presented in Table 3.

**Table 3:** Clinical variables associated with cirrhosis in adults and children under 16 with CFLD: Results of logistic regression analysis.

Clinical Variable	No Cirrhosis	Cirrhosis	Odds Ratio (OR)	OR 95% Confidence Interval	p-value
**UNIVARIATE ANALYSIS**					
**Children (<16 years)** (n = 1062)					
*Age* (n = 1062)					
0-9yrs	493 (49.8%)	15 (20.8%)			
9-16yrs	497 (50.2%)	57 (79.2%)	3.77	2.11 to 6.75	<0.0001
*Sex* (n = 1062)					
Female	443 (44.7%)	31 (43.1%)			
Male	547 (55.3%)	41 (56.9%)	1.07	0.66 to 1.74	0.78
*DF508 mutation* (n = 1016)					
0 copies	58 (6.1%)	10 (14.3%)			0.031
1 copy	315 (33.3%)	22 (31.4%)	0.40	0.18 to 0.90	0.026
2 copies	573 (60.6%)	38 (54.3%)	0.39	0.18 to 0.81	0.012
*Ursodeoxycholic acid therapy* (n = 1024)	13 (18.8%)	56 (81.2%)	2.87	1.55 to 5.32	0.001
*Diabetes* (n = 1062)	32 (3.2%)	7 (9.7%)	3.22	1.37 to 7.59	0.007
*Chronic P*. *aeruginosa infection* (n = 1062)					
No	623 (62.9%)	41 (56.9%)			
Yes	367 (37.1%)	31 (43.0%)	2.13	1.16 to 3.89	0.014
**Adults** (n = 2355)					
*Sex* (n = 2355)					
Female	845 (40.7%)	95 (33.8%)			
Male	1229 (59.3%)	186 (66.2%)	1.35	1.04 to 1.75	0.026
*BMI* (n = 2298)					
Mean (±SD)	21.98 ± 3.65	21.39 ± 3.70	0.95	0.92 to 0.99	0.012
<18.5kg/m^2^	287 (14.2%)	51 (18.8%)			0.041
18.5–25 kg/m^2^	1381 (68.2%)	189 (69.5%)	0.77	0.55 to 1.08	0.126
25–30 kg/m^2^	303 (15.0%)	26 (9.6%)	0.48	0.29 to 0.80	0.004
30–35 kg/m^2^	41 (2.0%)	3 (1.1%)	0.4	0.12 to 1.38	0.150
>35 kg/m^2^	14 (0.7%)	3 (1.1%)	1.21	0.34 to 4.35	0.775
*FEV1* (n = 679					
>80%	594 (29.7%)	72 (26.8%)			0.103
60–80%	551 (27.6%)	70 (26.0%)	1.05	0.74 to 1.49	0.792
40–60%	482 (24.1%)	60 (22.3%)	1.03	0.71 to 1.48	0.886
<40%	371 (18.6%)	67 (24.9%)	1.49	1.04 to 2.13	0.029
*Ursodeoxycholic acid therapy* (n = 2247)	906 (45.7%)	214 (81.1%)	5.09	3.69 to 7.01	<0.0001
*Pancreatic enzyme use* (n = 2293)	1891 (93.4%)	262 (97.4%)	2.63	1.22 to 5.69	0.014
*Diabetes* (n = 2355)	734 (35.4%)	143 (50.9%)	1.89	1.47 to 2.43	<0.0001
*Chronic P*.*aeruginosa infection* (n = 2355)					
No	632 (30.5%)	209 (74.4%)			
Yes	1442 (69.5%)	72 (25.6%)	1.38	1.07 to 1.78	0.012
**MULTIVARIATE ANALYSIS**					
**Children (<16 years)** (n = 1062)					
*Ursodeoxycholic acid*			2.85	1.50 to 5.43	0.001
**Adults** (n = 2247)					
*Ursodeoxycholic acid*			5.12	3.71 to 7.06	<0.0001
*CF-related diabetes*			1.83	1.40 to 2.39	<0.0001
*Male sex*			1.60	1.20 to 2.12	0.001
*Chronic P*. *aeruginosa infection*			1.18	1.01 to 1.39	0.042

NB: Variables with clinical relevance or with p-values less than 0.05 in the univariate analysis were modelled together in the multivariate analysis

In children, older age (9 years to 16 years), having CF-related diabetes and chronic infection with *P*.*aeruginosa* were all significant variables on univariate analysis. There was no association between cirrhosis and male sex in children (OR 1.07 (95% CI 0.66–1.74), p = 0.78). In adults, male sex, chronic infection with *P*.*aeruginosa*, FEV1 <40%, CF-related diabetes and current pancreatic enzyme use (a surrogate marker of pancreatic insufficiency) were predictive of cirrhosis on univariate analysis.

On multivariate analysis, male sex, CF-related diabetes and chronic *P*.*aeruginosa* infection were significant independent factors associated with the presence of cirrhosis in the adult cohort. Distal intestinal obstruction syndrome (DIOS), lower weight, lower FEV_1_, smoking status and parent or patient education levels were not associated with cirrhosis.

### Factors predicting progression to cirrhosis in CFLD

**Fig 1:**
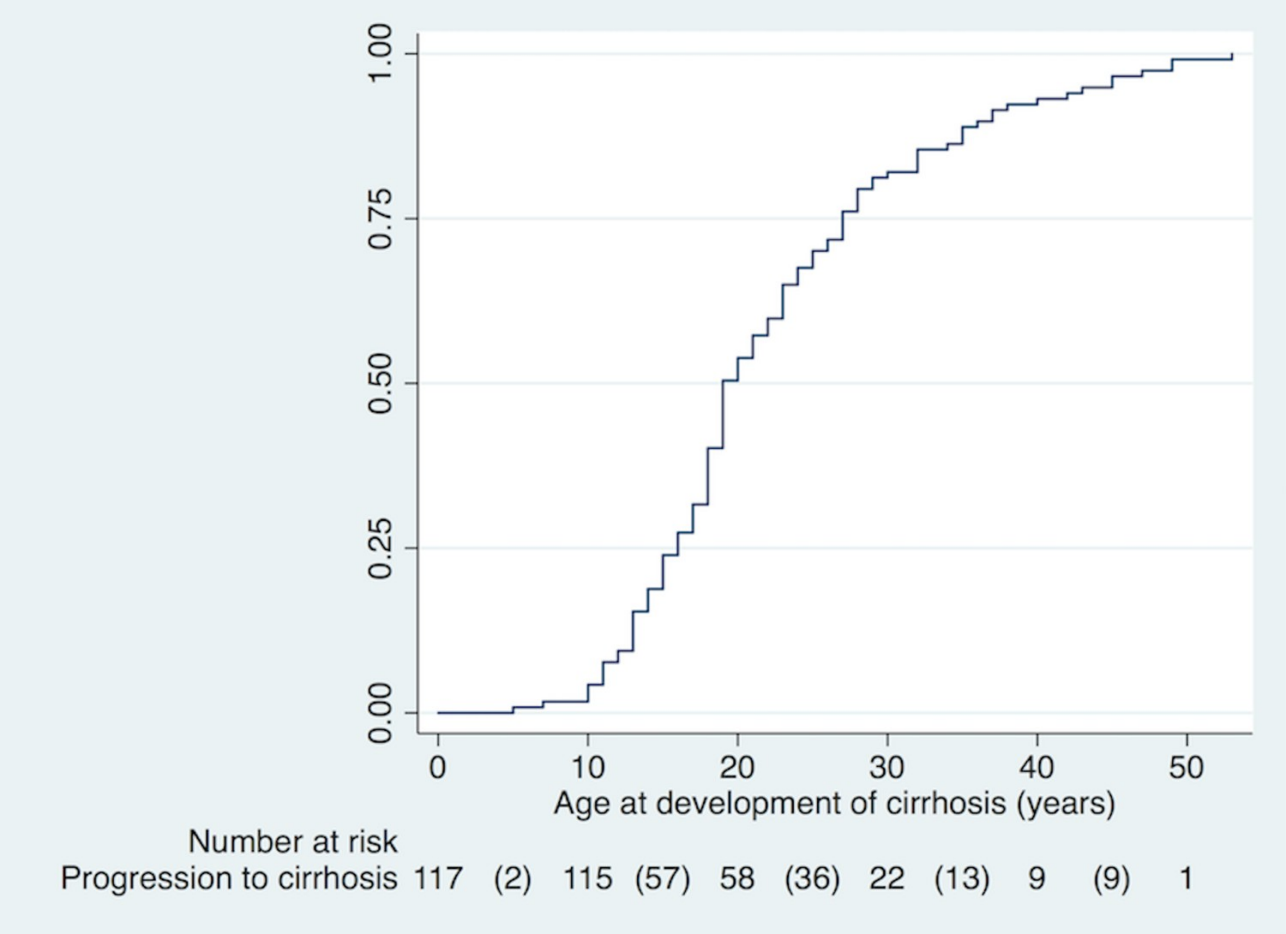
Cumulative frequency of progression to cirrhosis in patients with CFLD, by age (years). 117 CFLD patients developed progressive disease and liver cirrhosis during the study, at a median age of 19 years (IQR 16 to 27 years).

Variables associated with disease progression were determined using prospective follow-up of all subjects with CFLD who did not have cirrhosis at baseline (n = 3064). 117 (3.8%) progressed to cirrhosis during the study period. Of these, 44 were under 16 years and 73 were adults. During follow up, age at cirrhosis diagnosis ranged from 5 to 53 years, with a median age of 19 years (IQR 16 to 27 years). Cumulative frequency of disease progression to cirrhosis by age at time of progression is shown in Fig 1.

Cox proportional hazards modelling determined that CF-related diabetes (HR 1.88, 95% CI 1.25–2.84, p = 0.002) and an abnormal BMI were independent predictors of cirrhotic progression. BMI <18.5kg/m^2^ was independently associated with progression to cirrhosis (HR 1.76, 95% CI 1.16–2.68, p = 0.009), whilst BMI >25 kg/m^2^ also trended towards, but fell short of the significance threshold (HR 1.64, 95% CI 0.93–2.89, p = 0.086).

### 3.2 Factors predicting overall survival in CFLD

**Fig 2:**
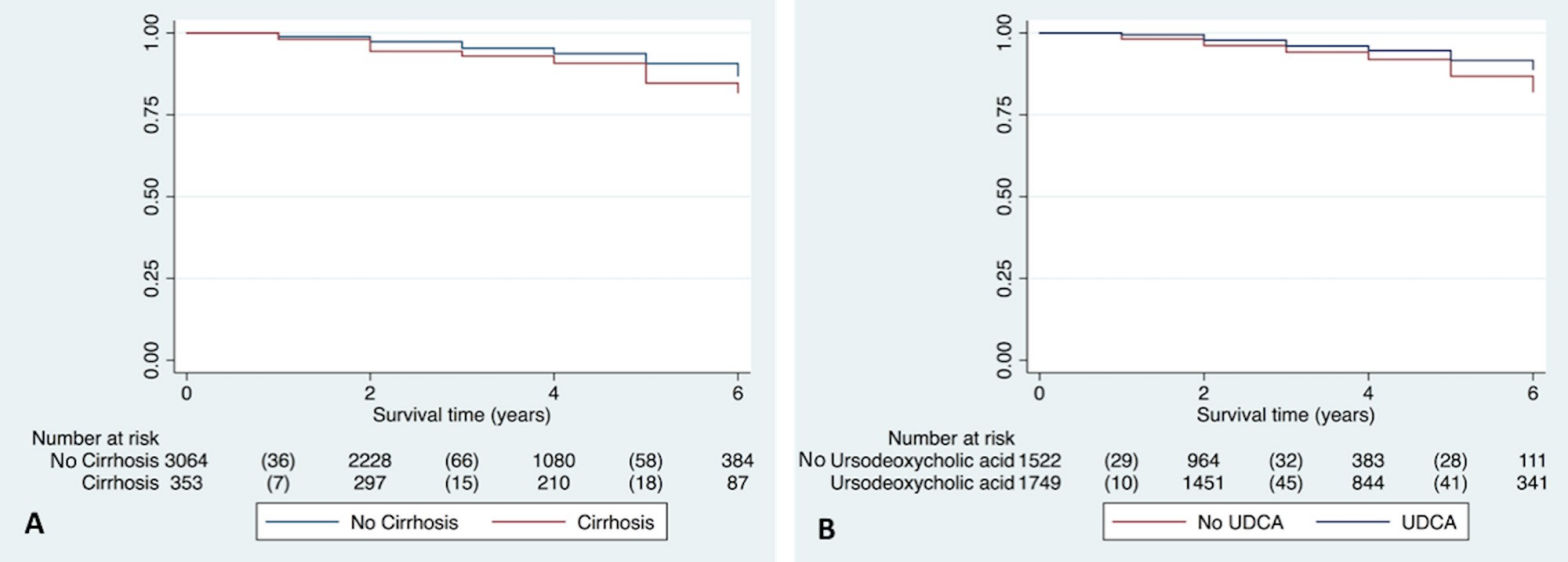
Survival in CFLD patients with and without cirrhosis and with and without ursodeoxycholic acid therapy. **A.** Survival was significantly shorter in patients with cirrhosis compared to those without cirrhosis (HR 1.54, 95% CI 1.09 to 2.18, p = 0.015). **B.** Survival was significantly longer in patients on ursodeoxycholic acid (HR 0.70, 95% CI 0.52–0.96, p = 0.026).

Overall (all-cause) survival time was compared between cirrhotic and non-cirrhotic CFLD patients. Survival was significantly shorter in patients with cirrhosis compared to those without (HR 1.54, 95% CI 1.09 to 2.18, p = 0.015; Fig 2A).

Analysis of variables associated with overall survival in all CFLD patients was undertaken. Cox proportional hazards modelling identified an FEV_1_ <60% (HR 5.33, 95% CI 3.54 to 8.04, p<0.0001), CF-related diabetes (HR 1.80, 95% CI 1.32–2.46, p<0.0001), age 16 years or older (HR 3.97, 95% CI 1.22–12.92, p = 0.022) and chronic infection with *P*.*aeruginosa* (HR 1.77, 95% CI 1.21–2.58, p = 0.003) as significant independent risk factors for reduced overall survival. By contrast, UDCA therapy was associated with prolonged overall survival (HR 0.70, 95% CI 0.52–0.96, p = 0.026; Fig 2B).

Stratification of CFLD patients by the presence of cirrhosis identified that UDCA use was associated with prolonged survival, specifically in those without cirrhosis (HR 0.50, 95% CI 0.36–0.69, p<0.0001) but not in those with cirrhotic disease (HR 1.19, 95% CI 0.46–3.10, p = 0.71). Of the patients not on UDCA, 1459 (95.9%) had non-cirrhotic liver disease and 63 (4.1%) had cirrhosis (S3 Table).

## 4. Discussion

This is the first UK nationwide study of CFLD and one of the largest multi-centre studies of its kind in Europe[5,7,9,12,14,15,17–19]. We have demonstrated that CFLD prevalence in the UK is slowly increasing and is associated with higher mortality compared to CF patients without liver disease. Our data suggest cirrhosis can be diagnosed in adulthood and also confirmed reports that male sex, *P*.*aeruginosa* and CF related diabetes are significant independent risk factors for cirrhosis in CF patients. Our data also suggest a possible survival benefit for ursodeoxycholic acid in CFLD.

### 4.1 Incidence, prevalence and mortality

Our reported incidence rate (63.5 cases per 1000 CF patients) was significantly higher than previous estimates [8,9]. Possible explanations for this include a more inclusive definition of CFLD than previous studies: patients with isolated radiological evidence of steatosis could be recorded in the ‘non-cirrhotic CFLD’ category. Such cases may reflect unrelated essential fatty acid deficiency[20]. Inclusion in the category of ‘elevated liver enzymes’ requires only one result above the upper limit of normal (either AST, ALT or GGT) in the preceding 12 months. It was not possible to determine from registry data if there were single or multiple elevated results in that period, and enzymes can be intermittently elevated for unrelated reasons including antibiotic use. Recently agreed diagnostic criteria do, however, include intermittent elevations in liver enzymes within non-cirrhotic disease[16].

Incidence rates may be overestimated in earlier years because the completeness of the denominator data used (number of patients at risk) improved during the study period: the percentage of complete records increased from 71% to 95% between 2008 and 2016[2]. Alternatively, higher incidence rates may be the result of improved detection from more frequent abdominal ultrasonography: this is recommended in alternate years in all children with CF in the UK; European and US guidelines specify ultrasonography only if there is clinically evident hepatomegaly[21–23].

Estimates of the prevalence of CFLD vary from 2% to 37%[7,8,12,14,24], likely reflecting a lack of coherent diagnostic criteria. Our results demonstrated an increase in prevalence between 2008 and 2013. The same inclusion criteria were used throughout and thus any increase is likely to be accurate. It is not possible from this data to identify the reasons for this increase in prevalence; however, the overall increase in life expectancy in patients with CF, in addition to improved detection through routine ultrasound surveillance, is likely to be contributory.

Previous reports on mortality in CFLD have provided contradictory results. Colombo et al. reported no increase in all-cause mortality in CFLD[9], whilst an earlier study by Scott-Jupp et al. reported a median life expectancy of 13.9 years in CFLD, much lower than those without liver disease[7]. A French cohort study showed that liver disease was an independent risk factor for lung transplantation or death[17]. In our study, CFLD was associated with a higher all-cause crude mortality rate compared to those with CF alone. In all, 24 patients underwent liver transplantation during the study period. Cirrhosis was associated with higher all-cause mortality and shorter overall survival compared with non-cirrhotic CFLD. The majority of deaths (97.5%) were in adults and secondary to non-hepatic complications (96%). This fact is not surprising, as liver cirrhosis is a systemic syndrome. Contributory factors include (but are not limited to) an increased susceptibility to infection[25], sarcopaenia[26,27], renal dysfunction[28] and cardiomyopathy[29]; all of which can render patients more vulnerable to cardiorespiratory failure during infectious exacerbations of their respiratory disease.

### 4.2 Risk factors in CF liver disease

There is conflicting evidence on the role of sex in CFLD. A number of longitudinal studies have demonstrated male preponderance both in CFLD as a whole[9] and specifically in more advanced disease with portal hypertension[15]. However other studies have reported no significant difference between sexes[8,30] and even a female preponderance in a cohort with biopsy proven liver disease[5]. These discrepancies could be a reflection of smaller sample sizes. In this large, population-level study of over 3000 cases we too report a male preponderance in CFLD. On regression analysis, male sex increased the risk of cirrhosis in adults with CFLD, but not children. This may reflect an ongoing incidence of cirrhosis in males, but not females, after puberty and would agree with the cumulative incidence data reported elsewhere[9]. A mechanistic explanation for this has yet to be identified, however a protective role of oestrogens has been suggested[31].

The independent association of *P*. *aeruginosa* infection with cirrhosis in CFLD may be the result of an increased susceptibility to infection in cirrhosis. Cirrhosis associated immune dysfunction (CAID) is a well described phenomenon in which immune cells demonstrate impaired clearance of pathogens and antimicrobial responses[25]. This is due to a number of reasons, including increased bacterial translocation from the GI tract in the context of portal hypertension and impaired antigenic clearance by Kupffer cells, the principal hepatic phagocyte. This results in vulnerability to overwhelming infection[32,33]. Haemodynamic changes as a consequence of advanced cirrhosis may permit the proliferation of *P*. *aeruginosa* species: hepatopulmonary syndrome in advanced cirrhosis is characterised by an exacerbation of pulmonary ventilation-perfusion mismatch and hypoxia[34]. A recent study showed that hypoxic conditions favoured the proliferation of resistant strains of *P*. *aeruginosa*[35].

The role of meconium ileus and DIOS in CFLD has been a subject of debate[7,8,36], however no evidence for an association was found in this large study. There was also no association between socioeconomic factors and disease severity in this study.

### 4.3 Factors affecting disease progression in CFLD

Our data suggest that progression to cirrhosis remains an ongoing issue in a significant proportion of CFLD patients, even into adulthood. This contradicts observations from previous longitudinal studies in which clinically evident CFLD primarily develops before puberty[5,9].

‘Adult-onset’ disease may represent a distinct, less severe form of CFLD. There is increasing evidence in the literature of such a phenomenon: a recent longitudinal study with up to 38 years of serial, multimodal follow-up has indeed identified cases of adult-onset disease[37] and a more benign disease phenotype has been previously described[38]. This disease may follow a similar natural history to other biliary cirrhotides, with a long pre-clinical phase. With an increasing life expectancy, we may see more hepatic decompensation later in life. However, we cannot exclude delayed diagnosis of ‘conventional’ CFLD contributing to the proportion of adult diagnoses in our study.

The ongoing incidence of cirrhosis into adulthood may reflect the impact of other risk factors for liver disease. Such ‘adult onset’ cirrhosis may commence with fatty change and steatohepatitis through parallel mechanisms with the rest of the adult UK population[39]. Of note, alcohol intake is not recorded on the UK registry. This lifestyle factor is a key risk modifier that we were unable to interrogate in this study.

In adults we have identified low BMI (≤ 18 kg/m^2^) as significantly associated with cirrhotic progression. Poor nutritional state may reflect the impact of progressive liver disease; nevertheless this finding would support the established concepts that optimal nutrition in both chronic liver disease and cystic fibrosis is essential to mitigate morbidity.

### 4.4 Ursodeoxycholic acid has a survival benefit in CFLD

This study suggests a survival benefit for ursodeoxycholic acid in CFLD, specifically in those without cirrhotic disease. Benefits for those with cirrhosis need further investigation in a larger sample with additional follow up. Although the survival effect was modest and over a short follow-up period, such an observation from this large dataset warrants further investigation and echoes the results of a small randomised controlled trial[40]. The role of UDCA in the management of CFLD has long been an area of debate: a recent cohort study and systematic review by the Cochrane collaboration did not recommend its use[19,41].

We compared CFLD patients who were on UDCA with those who were not: patients taking UDCA were more likely to have advanced liver disease (as would be expected) but are also younger and have better lung function. These findings may reflect prescribing practices: UDCA will have been more commonly prescribed once liver disease was overt. Nevertheless, our analyses highlight the need for a larger multi-centre randomised controlled trial of UDCA in CFLD to authoritatively address this clinical question.

Limitations with the data must be acknowledged. With the exception of mortality data, our analysis does not include patients without liver disease: we did not undertake comparative analysis with the remainder of the CF population. The Registry database entry categories for liver disease allow for the inclusion of patients with other aetiologies of liver disease (including viral hepatitis) under the broad heading of ‘non-cirrhotic liver disease’. As a result, the specific impact of viral hepatitis cannot be examined independently. In addition, there is some debate as to whether isolated steatosis or deranged liver function tests reflects true CFLD[5,22]. Recurrent antibiotic therapy and essential fatty acid malabsorption are well-known causes of these findings, and such fatty change is reversible and unrelated to progressive liver disease. However, it should be noted that clinical data for the registry are collected during a period of clinical stability so the effect of acute antibiotic courses on liver function will have been minimised.

The definitions of ‘liver disease’ in these patients is based on data entry by trained clinical staff, however the process is not necessarily overseen by a gastroenterologist/hepatologist. Furthermore, the diagnosis of cirrhosis in these patients is primarily radiological. Liver biopsy is not routinely performed in the UK and as a result, some patients with early cirrhotic change may be missed, thereby underestimating its true prevalence. Routine ultrasonography does, however, allow for accurate monitoring of disease progression. Further evaluation with non-invasive measurements of liver fibrosis (including transient elastography and magnetic resonance imaging) could play a pivotal role in screening in the future. Finally, accurate determination of the duration of follow-up was not possible as data was taken from sequential annual datasets without specific dates such as entry into the registry and date of diagnosis.

In spite of these limitations, this dataset is the largest of its kind describing CFLD and contains the data for >99% of UK CF patients. A robust system of auditing and cross-checking by a data management team ensures a high quality and comprehensive dataset. Our findings suggest new insights into both the natural history of CFLD and the role of ursodeoxycholic acid in CFLD. Further studies, with longer periods of follow-up and including those without liver disease are now required to verify these findings.

## Supporting information

S1 Table Summary of distribution of socio-economic variables across the study cohort. (DOCX)

S2 Table Annual crude mortality rates for CF patients and CFLD patients. (DOCX)

S3 Table Comparison of clinical features between patients with cystic fibrosis-related liver disease who were or were not prescribed ursodeoxycholic acid therapy. (DOCX)
