# Supplementary material for: The emerging burden of liver disease in cystic fibrosis patients: A UK nationwide study
Source: PLoS One. 2019 Apr 4;14(4):e0212779. doi: 10.1371/journal.pone.0212779 (PMC6448894; doi:10.1371/journal.pone.0212779)
Supplement: S1 Table — (DOCX) [file pone.0212779.s001.docx]

**S1 Table: Summary of distribution of socio-economic variables across the study cohort**

| **Clinical Variable** | **Number (%)/ Mean +/- SD** |
| --- | --- |
| Cigarette smoker (n=2932) | 63 (2.2%) |
| Patient education level (n=1753) |  |
| Less than high school | 658 (37.5%) |
| High school | 385 (22.0%) |
| Some college | 172 (9.8%) |
| College graduate | 228 (13.0%) |
| University | 310 (17.7%) |
| Patient employment (n=2263) |  |
| Under 15 years/ NA | 835 (36.9%) |
| Full time | 626 (27.7%) |
| Part time | 237 (10.5%) |
| Homemaker | 95 (4.2%) |
| Unemployed | 445 (19.7%) |
| Retired | 25 (1.1%) |
