# Supplementary material for: The emerging burden of liver disease in cystic fibrosis patients: A UK nationwide study
Source: PLoS One. 2019 Apr 4;14(4):e0212779. doi: 10.1371/journal.pone.0212779 (PMC6448894; doi:10.1371/journal.pone.0212779)
Supplement: S2 Table — (DOCX) [file pone.0212779.s002.docx]

**S2 Table: Annual crude mortality rates for CF patients and CFLD patients**

| **Year** | **Deaths CF patients without liver disease/ year** | **Death rate: CF patients without liver disease (per 1000 CF patients, per year)** | **CFLD patient deaths per year** | **Death rate CFLD patients (per 1000 CF patients, per year)** | **Comparative Mortality Figure (CMF)** |
| --- | --- | --- | --- | --- | --- |
| 2008 | 25/4845 | 5.2 | 21/1237 | 17.0 | 3.3 |
| 2009 | 63/5854 | 10.8 | 31/1527 | 20.3 | 1.9 |
| 2010 | 49/6317 | 7.8 | 18/1619 | 11.1 | 1.4 |
| 2011 | 58/6742 | 8.6 | 20/1938 | 10.3 | 1.2 |
| 2012 | 41/6823 | 6.0 | 38/1978 | 19.2 | 3.2 |
| 2013 | 76/7018 | 10.8 | 72/2067 | 34.8 | 3.2 |
| **Total*** | **365/47924***  **person-years** | **7.6** | **200/10366***  **person-years** | **19.3** | **2.5** |

* For the total six year study period, mortality rates were calculated per person-years at risk
