# Supplementary material for: The emerging burden of liver disease in cystic fibrosis patients: A UK nationwide study
Source: PLoS One. 2019 Apr 4;14(4):e0212779. doi: 10.1371/journal.pone.0212779 (PMC6448894; doi:10.1371/journal.pone.0212779)
Supplement: S3 Table — (DOCX) [file pone.0212779.s003.docx]

**S3 Table: Comparison of clinical features between patients with cystic fibrosis-related liver disease who were or were not prescribed ursodeoxycholic acid therapy (n=3271)**

| **Clinical Variable** | **Not on ursodeoxycholic acid** | **On Ursodeoxycholic acid** | **P-value** |
| --- | --- | --- | --- |
| Cirrhosis |  |  |  |
| No | 1459 (95.9%) | 1479 (84.6%) |  |
| Yes | 63 (4.1%) | 270 (15.4%) | <0.0001 |
| Stage of liver disease |  |  |  |
| Non-cirrhotic liver disease | 1459 (95.8%) | 1479 (84.5%) |  |
| Cirrhosis (no portal hypertension) | 24 (1.6%) | 124 (7.1%) |  |
| Cirrhosis with portal hypertension | 39 (2.6%) | 146 (8.4%) | <0.0001 |
| Gender |  |  |  |
| Male | 915 (60.1%) | 1010 (57.8%) |  |
| Female | 607 (39.9%) | 739 (42.2%) | 0.169 |
| Child (< 16 years) | 395 (26.0%) | 629 (36.0%) |  |
| Adult | 1127 (74.0%) | 1120 (64.0%) | <0.0001 |
| Mean age (years, +/- SD) | 23.3 +/- 12.9 years | 19.6 +/- 9.9 years | <0.0001 |
| FEV1 (%) |  |  |  |
| >80% | 442 (34.1%) | 615 (39.9%) |  |
| 60-80% | 357 (27.6%) | 435 (28.2%) |  |
| 40-59.99% | 262 (20.2%) | 296 (19.2%) |  |
| <40% | 234 (18.1%) | 197 (12.8%) | <0.0001 |
| Cystic fibrosis related diabetes |  |  |  |
| No | 1115 (73.3%) | 1284 (73.4%) |  |
| Yes | 407 (26.7%) | 465 (26.6%) | 0.921 |
| Chronic infection Pseudomonas |  |  |  |
| No/ scant growth | 909 (59.7%) | 1052 (60.1%) |  |
| Heavy growth | 613 (40.3%) | 697 (39.9%) | 0.805 |
| Death | 89 (5.6%) | 96 (5.5%) | 0.658 |
